# Onasemnogene Abeparvovec for Treating Pre-symptomatic Spinal Muscular Atrophy: An External Assessment Group Perspective of the Partial Review of NICE Highly Specialised Technology Evaluation 15

**DOI:** 10.1007/s41669-023-00439-6

**Published:** 2023-09-20

**Authors:** Marty Chaplin, Rebecca Bresnahan, Nigel Fleeman, James Mahon, Rachel Houten, Sophie Beale, Angela Boland, Yenal Dundar, Ashley Marsden, Pinki Munot

**Affiliations:** 1 Liverpool Reviews and Implementation Group, University of Liverpool, Whelan Building, Brownlow Hill, Liverpool, L69 3GB UK; 2 Coldingham Analytical Services, Berwickshire, UK; 3 Hare Research, North Yorkshire, UK; 4 North West Medicines Information Centre, Liverpool, UK; 5 Great Ormond Hospital for Children NHS Foundation Trust, London, UK

## Abstract

As part of the National Institute for Health and Care Excellence (NICE) highly specialised technology (HST) evaluation programme, Novartis submitted evidence to support the use of onasemnogene abeparvovec as a treatment option for patients with pre-symptomatic 5q spinal muscular atrophy (SMA) with a bi-allelic mutation in the survival of motor neuron *(SMN)* 1 gene and up to three copies of the *SMN2* gene. The Liverpool Reviews and Implementation Group at the University of Liverpool was commissioned to act as the External Assessment Group (EAG). This article summarises the EAG’s review of the evidence submitted by the company and provides an overview of the NICE Evaluation Committee’s final decision, published in April 2023. The primary source of evidence for this evaluation was the SPR1NT trial, a single-arm trial including 29 babies. The EAG and committee considered that the SPR1NT trial results suggested that onasemnogene abeparvovec is effective in treating pre-symptomatic SMA; however, long-term efficacy data were unavailable and efficacy in babies aged over 6 weeks remained uncertain. Cost-effectiveness analyses conducted by the company and the EAG (using a discounted price for onasemnogene abeparvovec) explored various assumptions; all analyses generated incremental cost-effectiveness ratios (ICERs) that were less than £100,000 per quality-adjusted life-year (QALY) gained. The committee recommended onasemnogene abeparvovec as an option for treating pre-symptomatic 5q SMA with a bi-allelic mutation in the *SMN1* gene and up to three copies of the *SMN2* gene in babies aged ≤ 12 months only if the company provides it according to the commercial arrangement (i.e. simple discount patient access scheme).

**Supplementary Information:**

The online version contains supplementary material available at 10.1007/s41669-023-00439-6.

## Key Points for Decision Makers

**Table:** 

Trial evidence supporting the use of onasemnogene abeparvovec as a treatment for patients with pre-symptomatic spinal muscular atrophy was only available from one small, single-arm trial (SPR1NT trial, *n* = 29).
It is not yet known whether patients treated pre-symptomatically with onasemnogene abeparvovec will maintain their achieved motor milestones for life.
Cost-effectiveness analyses conducted by the company and the External Assessment Group used a discounted price for onasemnogene abeparvovec and explored a range of different assumptions; all analyses generated incremental cost-effectiveness ratios that were less than £100,000 per quality-adjusted life-year gained.

## Introduction

The National Institute for Health and Care Excellence (NICE) is an independent organisation responsible for providing guidance to the National Health Service (NHS) in England and Wales on a range of clinical and public health issues, as well as appraisals of new health technologies. In the NICE highly specialised technology (HST) evaluation programme, recommendations are made on the use of new and existing highly specialised medicines and treatments within the NHS in England and Wales. An HST evaluation focuses on a single technology for a single indication and is typically used for new technologies close to launch in the UK [1]. The evidence for an HST evaluation is principally derived from a submission by the company that manufactures the technology and is based on a specification developed by NICE. An external, independent organisation (typically, an academic group) known as the External Assessment Group (EAG), provides a critique of the company’s submission (the EAG report). Consultees, clinical specialists and patient representatives also provide additional information during the appraisal process.

Using a specification developed by NICE (the final scope), the NICE Highly Specialised Technologies Evaluation Committee (HSTEC) meet to consider the company’s submission, the EAG report, and testimonies from experts and stakeholders to determine whether the technology represents a clinically effective and cost-effective use of NHS resources. The committee then makes recommendations about whether the technology should be provided by the NHS. If the committee’s recommendations are restrictive, preliminary guidance is issued in the form of an evaluation consultation document (ECD). All stakeholders and the public have an opportunity to comment on the ECD, before the committee meets again to consider the comments and produce the final evaluation determination (FED). If the recommendations are not restrictive, the ECD is not required, and the committee produces the FED following the first committee meeting. The final guidance, if positive, constitutes a legal obligation for NHS providers in England and Wales to make the technology available in line with NICE’s recommendations.

This article presents a summary of the EAG report for the partial review of NICE HST15, which evaluated onasemnogene abeparvovec for treating spinal muscular atrophy (SMA) [2]. Full details of relevant documents (including the scope, EAG report, company and consultee submissions, NICE guidance and comments on each of these) are available on the NICE website for HST15 [2], and the partial review of HST15 (identified on the NICE website as HST24) [3].

## The Decision Problem

### Underlying Health Problem

SMA is a rare genetic neuromuscular disorder characterised by muscle weakness and progressive loss of motor function [4]. The focus of HST15 [2] and the partial review of HST15 [3] was 5q SMA, which is caused by a bi-allelic mutation in the survival of the motor neuron 1 (*SMN1*) gene located in chromosome 5q. All references to SMA hereafter are to 5q SMA, which accounts for 95% of SMA cases. The bi-allelic mutation results in a lack of the survival of motor neuron (SMN) protein, which is necessary for normal motor neuron function, leading to motor neuron degeneration [4]. Patients with SMA experience substantial disability and, in many cases, reduced life expectancy [4, 5].

The survival of motor neuron 2 (*SMN2*) gene produces low levels of functional SMN protein, which can partially compensate for a bi-allelic mutation in the *SMN1* gene. In general, the higher the number of copies of the *SMN2* gene, the less severe the disease phenotype [6]. Clinically, SMA is classified depending on disease severity, which ranges from type 0 SMA (the most severe disease phenotype) to type 4 SMA (the least severe disease phenotype) [7]. A summary of the key features of SMA types is provided in Table 1.

**Table 1:** Key features of SMA types

SMA type	Description used in CS	Age at symptom onset	Highest motor milestone achievable	Life expectancy (BSC only)
0	NA	Pre-natal or at birth	Nil, require respiratory support from birth	Days to weeks
1	Non-sitter	< 6 months^a^	Unable to sit without support Over time, lose the ability to swallow and experience respiratory complications, ultimately resulting in death from respiratory failure	< 2 years (without ventilatory support)
2	Sitter	6–18 months	Able to sit without support (normally outside the normal developmental window) Some babies may crawl and stand alone, but do not achieve walking alone Upon disease progression, may lose previously achieved motor milestones	20–60 years
3	Walker	1.5–10 years	Able to walk May lose the ability to walk alone and stand alone after symptom onset	Normal
4	NA	> 35 years	Able to walk May develop reduced mobility after symptom onset	Normal

Most patients (95.7%) with two copies of the *SMN2* gene develop type 1 SMA, and most patients with three copies of the *SMN2* gene develop type 2 (54.3%) or type 3 (30.9%) SMA (Table 2). Source: Adapted from NICE [8], Table 3

*BSC* best supportive care, *CS* company submission, *EAG* External Assessment Group, *NA* not applicable, *SMA* spinal muscular atrophy

^a^Clinical advice to the EAG was that babies with type 1 SMA present with symptoms between age 4 and 6 weeks and are normally clinically diagnosed between age 8 and 12 weeks

**Table 2:** Expected SMA type by number of copies of the *SMN2* gene

*SMN2* gene copies	SMA type			
	Type 1 [*n* = 1256]	Type 2 [*n* = 1160]	Type 3 [*n* = 1017]	Type 4 [*n* = 26]
1	95.7	4.3	0.0	0.0
2	78.9	16.5	4.5	0.1
3	14.7	54.3	30.9	0.1
≥ 4	0.7	11.5	83.3	4.4

Data are expressed as percentages. Source: EAG report, Table 2, adapted from Calucho et al. [6]

*SMA* spinal muscular atrophy, *SMN2* survival motor neuron 2

Approximately 60 babies are born with SMA each year in England and around 60% of these are clinically diagnosed with type 1 SMA [9].

### Intervention

Onasemnogene abeparvovec, a one-time gene replacement therapy delivered by intravenous infusion, addresses the underlying genetic cause of SMA. It is indicated for the treatment of patients with SMA with a bi-allelic mutation in the *SMN1* gene and a clinical diagnosis of type 1 SMA, or patients with SMA with a bi-allelic mutation in the *SMN1* gene and up to three copies of the *SMN2* gene [10].

### Partial Review of Highly Specialised Technology Evaluation 15

On completion of HST15 [2] in July 2021, NICE made the recommendations outlined in Table 3.

**Table 3:** NICE HST15 recommendations

Recommendation	
1.1	Onasemnogene abeparvovec is recommended as an option for treating 5q SMA with a bi-allelic mutation in the *SMN1* gene and a clinical diagnosis of type 1 SMA in babies, only if: • they are 6 months or younger, or • they are aged 7–12 months and their treatment is agreed by the national multidisciplinary team It is only recommended for these groups if: • permanent ventilation for more than 16 h per day or a tracheostomy is not needed • the company provides it according to the commercial arrangement
1.2	For babies aged 7–12 months, the national multidisciplinary team should develop auditable criteria to enable onasemnogene abeparvovec to be allocated to babies in whom treatment will give them at least a 70% chance of being able to sit independently
1.3	Onasemnogene abeparvovec is recommended as an option for treating pre-symptomatic 5q SMA with a bi-allelic mutation in the *SMN1* gene and up to three copies of the *SMN2* gene in babies. It is recommended only if the conditions in the managed access agreement are followed

Source: HST15 [2]

*NICE* National Institute for Health and Care Excellence, *SMA* spinal muscular atrophy, *SMN1* survival motor neuron 1, *SMN2* survival motor neuron 2

The current evaluation (partial review of HST15) focused on recommendation 1.3. The company presented new evidence, which was not available at the time of the original evaluation, to support the use of onasemnogene abeparvovec as a treatment option for patients with pre-symptomatic 5q SMA with a bi-allelic mutation in the *SMN1* gene and up to three copies of the *SMN2* gene.

A pre-symptomatic diagnosis of SMA requires genetic testing; there is currently no UK national screening programme for SMA [11]. Only babies who have a sibling with SMA or a parent with confirmed carrier status are genetically tested for SMA; this testing identifies approximately two babies with pre-symptomatic SMA and up to three copies of the *SMN2* gene each year [12]. An ongoing UK population-based pilot study [13] is investigating the feasibility of using spare capacity from the NHS newborn blood spot screening programme to provide national screening for SMA. It is anticipated that the pilot study [13] will identify between one and three additional patients with pre-symptomatic SMA and up to three copies of the *SMN2* gene each year [14].

The final scope [15] developed by NICE for the partial review of HST15 listed best supportive care (BSC) as the appropriate comparator for this evaluation. The aim of BSC is to manage SMA upon symptom onset by minimising disability and improving health-related quality of life (HRQoL). BSC does not prevent disease progression but may extend life [7, 16]. At the time of this evaluation, no active treatments for pre-symptomatic SMA were routinely commissioned in NHS clinical practice.

Following recommendations made by NICE in HST15 [2], onasemnogene abeparvovec became part of NHS clinical practice for patients with symptomatic type 1 SMA (see Table 3). Therefore, the EAG considered that the relevant comparison (hereafter referred to as the EAG’s preferred comparison) for the partial review of HST15 was:

- providing onasemnogene abeparvovec pre-symptomatically to the pre-symptomatic patient versus
- providing (1) onasemnogene abeparvovec, or (2) BSC only at symptom onset if (a) the baby develops type 1 SMA or (b) if the baby develops type 2 or 3 SMA.

## External Assessment Group Report

The evidence provided by the company comprised an initial submission, an economic model (which is commercial in confidence) and the company’s response to the EAG’s clarification requests [3]. The EAG report comprised a summary and critical review of the clinical and cost-effectiveness evidence provided by the company.

### Clinical Evidence

The primary source of clinical evidence presented by the company was the phase III, open-label, single-arm, multicentre SPR1NT trial, which assessed the efficacy of onasemnogene abeparvovec as a treatment for patients with pre-symptomatic SMA and two (*n* = 14) [17] or three (*n* = 15) [18] copies of the *SMN2* gene. The *SMN2* two-copy and *SMN2* three-copy cohorts had different primary and secondary efficacy endpoints and lengths of follow-up in the trial.

In the SPR1NT trial two-copy *SMN2* cohort [17], all 14 patients met the primary endpoint of functional independent sitting (as defined by the Bayley Scales of Infant and Toddler Development Gross Motor [BSID GM] subtest item number 26) at any visit up to age 18 months, and the secondary endpoint of event-free survival (i.e. survival without the need for permanent ventilation) at age 14 months. The majority (11/14, 78.6%) of patients achieved the primary endpoint within the normal development window (as defined by the World Health Organization Multicentre Growth Reference Study [WHO-MGRS]) [19]. All except one patient (13/14, 92.9%) met the secondary endpoint of weight maintenance at or above the third percentile (without non-oral/mechanical feeding support) up to age 18 months.

In the SPR1NT trial three-copy *SMN2* cohort [18], all 15 patients met the primary endpoint of standing alone (as defined by the BSID GM subtest item number 40) at any visit up to age 24 months, and 14 patients (93.3%) met the secondary endpoint of walking alone (as defined by the BSID GM subtest item number 43) at any visit up to age 24 months. Most patients achieved these milestones within the normal development windows (as defined by WHO-MGRS) [19] (standing alone: 14/15, 93.3%; walking alone: 11/15, 73.3%).

In the company’s submission for the partial review of HST15, the company presented evidence for the comparison of providing onasemnogene abeparvovec pre-symptomatically to the pre-symptomatic patient versus BSC. As the SPR1NT trial [17, 18] did not have a control arm, the company presented data from the Pediatric Neuromuscular Clinical Research (PNCR) dataset [20] to provide evidence of clinical outcomes among patients receiving BSC only. The company compared data from the SPR1NT trial two-copy *SMN2* cohort [17] with data from a cohort of patients in the PNCR dataset who had two copies of the *SMN2* gene and type 1 SMA (*n* = 23), and compared data from the SPR1NT trial three-copy *SMN2* cohort [18] with data from a cohort of patients in the PNCR dataset who had three copies of the *SMN2* gene and any SMA type (*n* = 81).

In response to a clarification request, the company provided an updated model that included cost-effectiveness evidence to support the EAG’s preferred comparison. However, the company did not provide clinical-effectiveness evidence to support this comparison, other than the information included in the updated model.

Therefore, to inform the EAG’s preferred comparison, the EAG carried out simple naïve comparisons of data from the SPR1NT trial [17, 18] versus data from the START [21], STR1VE-US [22] and STR1VE-EU [23] trials, which assessed onasemnogene abeparvovec as a treatment for patients with type 1 (symptomatic) SMA. The EAG also compared data from the SPR1NT trial [17, 18] with data from the PNCR [20] three-copy *SMN2* cohort. A subset of this cohort had type 2 or type 3 SMA, and therefore were relevant to the EAG’s preferred comparison.

Data from the SPR1NT [17, 18], START [21], STR1VE-US [22] and STR1VE-EU [23] trials and the PNCR [20] three-copy *SMN2* gene cohort for the primary and secondary outcomes of the SPR1NT trial are presented in Table 4. For completeness, the EAG also presented data from the PNCR two-copy *SMN2* cohort [20], as these data were used by the company to provide an external control arm for the SPR1NT trial [17].

**Table 4:** Comparison of key outcomes from the SPR1NT, STR1VE and START trials and the PNCR dataset

Outcome^a^		Pre-symptomatic SMA		Symptomatic SMA				
				Type 1			Type 1	Type 1, 2, 3
		Onasemnogene abeparvovec					BSC	
		SPR1NT [17] two-copy *SMN2* cohort [*n *= 14]	SPR1NT [18] three-copy *SMN2* cohort [*n *= 15]	START [21] cohort 2 [*n *= 12]^b^	STR1VE-US [22] [*n *= 22]^b^	STR1VE-EU [23] [*n *= 33]^b,c^	PNCR [20] two-copy *SMN2* cohort [*n *= 23]	PNCR [20] three-copy *SMN2* cohort [*n *= 81]
		Age 18 months	Age 24 months	24 months after first OA dose	Age 18 months	Age 18 months	18 months^d^	24 months^d^
Sits without support	≥ 30 s BSID GM item #26	14 (100.0)	14 (93.3)	9 (75.0)	14 (63.6)	16^e^ (48.5)	0	NR
Stands alone	≥ 3 s BSID GM item #40	11 (78.6)	15 (100.0)	2 (16.7)	1 (4.5)	1 (3.0)	0	19 (23.5)
Walks alone	≥ 5 steps with coordination and balance BSID GM item #43	9 (64.3)	14 (93.3)	2 (16.7)	1 (4.5)	1 (3.0)	0	17 (21.0)
Ability to maintain weight^f^ without the need for non-oral/mechanical feeding support at any visit		13 (92.9)	10 (66.7)	NR	14 (63.6)	15(65.2)^g^	NR	NR
Event-free survival at age 14 months^h^		14 (100)	15 (100)	NR	20 (90.9)	31 (96.9)^i^	6 (26.1)	Confidential data

Data are expressed as *n* (%). Source: EAG report [24], Table 17

*BSC* best supportive care, *BSID GM* Bayley Scales of Infant and Toddler Development (Version 3) Gross Motor subtest, *EAG* External Assessment Group, *ESM* electronic supplementary material, *ITT* intention to treat, *NR* not reported, *OA * onasemnogene abeparvovec, *PNCR* Pediatric Neuromuscular Clinical Research, *SMA* spinal muscular atrophy, *WHO* World Health Organization

^a^Outcome definitions for motor milestones (sits without support, stands alone, walks alone) used in the PNCR [20] dataset differed to those used in the onasemnogene abeparvovec trials. ESM Table 1 provides the definition of outcomes for the PNCR [20] dataset

^b^All patients in the START, STRIVE-US and STRIVE-EU trials had two copies of the *SMN2* gene

^c^Exploratory motor milestones in the STR1VE-EU [23] trial were assessed in the efficacy and safety completers population (*n* = 33)

^d^It is unclear whether data from the PNCR [20] dataset were reported for patients at age 18 months and 24 months, or at 18 months and 24 months follow-up from the time of enrolment

^e^Sits without support (BSID GM item #26) was also reported for the STR1VE-EU [23] ITT population (*n/N* = 14/32, 43.8%)

^f^Maintained weight consistent with age (above the third percentile for age and sex as defined by WHO guidelines)

^g^Reported as a proportion of ability to thrive population (patients who had intact swallowing and received no nutritional support at baseline, *n* = 23)

^h^Event-free survival defined as avoidance of both death and permanent ventilation through the 14 months of age visit. Permanent ventilation is defined as tracheostomy or the requirement of ≥16 h of respiratory assistance per day (via non-invasive ventilatory support) for ≥14 consecutive days in the absence of an acute reversible illness, excluding perioperative ventilation

^i^Assessed in the ITT population (*N* = 32)

Generally, outcomes for patients treated pre-symptomatically with onasemnogene abeparvovec were better than outcomes for patients who received onasemnogene abeparvovec upon clinical diagnosis of type 1 SMA, and were better than outcomes for patients who received BSC only for any type of SMA.

The company presented adverse event (AE) data from the SPR1NT trial [17, 18]. All patients (29/29, 100%) experienced at least one treatment-emergent AE (TEAE), most frequently pyrexia (18/29, 62.1%) and upper respiratory tract infection (14/29, 48.3%). Eighteen patients (62.1%) experienced at least one TEAE that was considered by the investigator to be related to treatment with onasemnogene abeparvovec. No patient experienced a TEAE that resulted in death or trial discontinuation.

Neither patient or carer HRQoL data were collected as part of the SPR1NT [17, 18], START [21], STR1VE-US [22] or STR1VE-EU [23] trials.

### Critique of the Clinical Evidence and Interpretation

The EAG considered that SPR1NT trial results suggested that onasemnogene abeparvovec is a clinically effective treatment for babies with pre-symptomatic SMA and two or three copies of the *SMN2* gene. However, clinical advice to the EAG was that uncertainty remains about the long-term efficacy of onasemnogene abeparvovec in clinical practice, particularly whether deterioration would occur. The company presented interim efficacy and safety data from a long-term study (LT-002) of patients with SMA (follow-up to age 15 years) treated with onasemnogene abeparvovec in clinical trials, but final results will not be available until the study’s completion in December 2035.

The EAG cautioned that the naïve comparisons of data from the SPR1NT, START [21], STR1VE-US [22] and STR1VE-EU [23] trials and the PNCR [20] dataset were not robust, as differences between trial and patient characteristics were not accounted for. For example, the mean age at symptom onset for patients in the PNCR [20] dataset (3.0 months) was greater than for patients in the START [21] (1.4 months), STR1VE-US [22] (1.9 months) and STR1VE-EU [23] (1.6 months) trials. Furthermore, sample sizes of the included trials and the PNCR dataset were all relatively small; this was expected given the rarity of SMA.

Evidence to inform the EAG’s preferred comparison was also limited as there was no evidence for the effectiveness of onasemnogene abeparvovec as a treatment for patients with type 1 SMA and three copies of the *SMN2* gene, as the START [21], STR1VE-US [22] and STR1VE-EU [23] trials only included patients with type 1 SMA and two copies of the *SMN2* gene. Furthermore, in the PNCR [20] dataset, the cohort of patients with three copies of the *SMN2* gene included some patients with type 1 SMA; in NHS clinical practice, patients with type 1 SMA may be eligible for [25] and receive treatment with onasemnogene abeparvovec, rather than BSC.

Clinical advice to the EAG was that safety data from all onasemnogene abeparvovec trials provides more comprehensive information than safety data collected from patients with pre-symptomatic SMA only. The EAG noted that safety data for 99 patients who received onasemnogene abeparvovec as a treatment for pre-symptomatic or symptomatic SMA at the recommended dose were reported in the European Medicines Agency (EMA) European Public Assessment Report (EPAR) [26]. The AEs most frequently reported from five open-label trials (SPR1NT, START [21], STR1VE-US [22], STR1VE-EU [23] and STR1VE-AP [27]), and described as very common (> 10%) or common (> 1%), were increased hepatic enzyme (24/99, 24.2%), hepatotoxicity (9/99, 9.1%), vomiting (8/99, 8.1%), thrombocytopenia (6/99, 6.1%), increased troponin (5/99, 5.1%), and pyrexia (5/99, 5.1%). The EPAR highlighted that outside clinical studies, including in the postmarketing setting, there had been reports of children experiencing thrombotic microangiopathy and developing signs and symptoms of acute liver failure.

The EAG also highlighted that more recently (11 August 2022), it was reported that two children out of more than 2300 patients worldwide who had been treated with onasemnogene abeparvovec experienced acute liver failure resulting in death [28]. These deaths were reported to occur between 5 and 6 weeks after onasemnogene abeparvovec infusion, and between 1 and 10 days after corticosteroid tapering occurred.

### Cost-Effectiveness Evidence

The company’s economic evaluation compared the cost effectiveness of onasemnogene abeparvovec versus BSC for treating patients with pre-symptomatic SMA and two or three copies of the *SMN2* gene. The company considered the population as a whole, with results weighted by number of copies of the *SMN2* gene. Subgroup analyses for the patient cohorts with two or three copies of the *SMN2* gene were carried out.

The company developed a two-part model (short- and long-term components) using Microsoft Excel (Microsoft Corporation, Redmond, WA, USA). The experience of patients receiving onasemnogene abeparvovec was modelled using the short-term component (61 months) and the long-term component (lifetime), while the experience of patients treated with BSC was modelled using only the long-term component.

The short-term component of the model was populated with data from the START [21], STR1VE-US [22] and STR1VE-EU [23] trials for patients with type 1 SMA and two copies of the *SMN2* gene. In the absence of data for patients with type 1 SMA and three copies of the *SMN2* gene, the company assumed that the efficacy of onasemnogene abeparvovec for these patients was the same as for patients with type 1 SMA and two copies of the *SMN2* gene.

The long-term component of the model had a time horizon of 100 years and a cycle length of 1 month. This Markov state-transition model comprised five mutually exclusive health states that reflected the highest motor function milestones achieved by patients. These health states were referred to by the company as: ‘HS1 (non-sitter, permanent assisted ventilation [PAV])’; ‘HS1 (non-sitter, no PAV)’; ‘HS2 (sitter)’; ‘HS3a (delayed walker)’; ‘HS3b (experiences later-onset SMA)’. Data from patients with type 1 (HS1), type 2 (HS2) and type 3 (HS3) SMA were used to populate these health states.

The time point at which patients entered a health state was estimated using the WHO [19] thresholds for sitting and walking. Patients who did not meet developmental milestones were moved to lower functioning health states. Patients could progress to death from any health state. Estimates for the proportions of untreated non-sitter patients requiring PAV were derived from Wijngaarde et al. [29] and from the NeuroNext [20] study. Milestone losses were estimated using data published by Wadman et al. [30].

Patients treated with onasemnogene abeparvovec entered the long-term model component in the same health state that was assigned to them in the short-term model component (based on motor function milestones achieved at the end of the SPR1NT trial and interim data from the LT-002 study [31]), where they remained until death. In the BSC arm, the distribution of patients between initial health states was informed by the distribution of patients across SMA type reported by Calucho et al. [6] (*n* = 3459), based on the proxy relationship between SMA type and motor milestone achievement.

Survival data sources used to populate the short-term and long-term model components were sourced from the SPR1NT trial and the LT-002 study [31], UK National Life tables (2018–2020) [32], an Italian natural history study [33], the NeuroNext study [5, 20] and Wijngaarde et al. [29].

Standard methods were used to fit parametric distributions to available data. To avoid clinically implausible survival estimates, curves were terminated based on observed life expectancy, clinical expert opinion, or assumptions that were preferred by the Evidence Review Group (ERG) in HST15 [2].

The utility values used to populate the model were sourced from the literature (Table 5).

**Table 5:** Company model utility values

Health state	Utility value	References
HS1 (non-sitter, PAV)	0	Interim ERG report; Edwards et al. [34] Thompson et al. [35]
HS1 (non-sitter, no PAV) and HS2 (sitter, loses sitting)	0.190	
HS2 (sitter)	0.600	Tappenden et al. [36]
HS3a (delayed walker)	General population	Ara and Brazier [37]
HS3b (experiences later-onset SMA)		
HS3a (delayed walker, loses walking) and HS3b (experiences later-onset SMA, loses walking)	0.774	Thompson et al. [35]
HS-BRND	General population	Ara and Brazier [37]

Source: EAG report [24], Table 32

*BRND* broad range of normal development, *EAG* External Assessment Group, *ERG* Evidence Review Group, *PAV* permanent assisted ventilation, *SMA* spinal muscular atrophy

The cost of treatment with onasemnogene abeparvovec was estimated based on the confidential discounted Patient Access Scheme (PAS) price. Health state costs were sourced from NHS Reference Costs 2019–2020 [38], the NHS Business Services Authority prescription cost analysis 2021/2022 [39] and the literature; where necessary, costs were inflated to 2021 prices using the National Health Service Cost Inflation Index (NHSCII) [40]. Costs associated with AEs were not included in the company model due to difficulties distinguishing between AEs due to treatment and SMA complications.

In response to a clarification request from the EAG, the company provided cost-effectiveness results to inform the EAG’s preferred comparison [providing onasemnogene abeparvovec pre-symptomatically versus providing (1) onasemnogene abeparvovec at symptom onset if the patient develops type 1 SMA, and (2) BSC at symptom onset for all other SMA types].

For the comparison of pre-symptomatic onasemnogene abeparvovec versus BSC, the company’s cost-effectiveness results suggested that the ICER per quality-adjusted life-year (QALY) gained was likely to be less than £100,000. For the comparison of pre-symptomatic onasemnogene abeparvovec versus onasemnogene abeparvovec on development of symptoms of type 1 SMA and BSC for all other types of SMA, the results suggested that pre-symptomatic treatment with onasemnogene abeparvovec was likely to be dominant.

### Critique of the Cost-Effectiveness Evidence and Interpretation

The EAG carried out a comprehensive check of the company model data inputs and algorithm and was satisfied that the model algorithms were accurate. The EAG was satisfied that the cost-effectiveness results generated by the company’s model were robust and suitable for decision making for both the company’s preferred comparison (providing onasemnogene abeparvovec pre-symptomatically versus BSC) and for the EAG’s preferred comparison [providing onasemnogene abeparvovec pre-symptomatically versus providing (1) onasemnogene abeparvovec only at symptom onset if the patient develops type 1 SMA, and (2) BSC at symptom onset for all other SMA types].

The company provided results for the whole population and independently for patients with two or three copies of the *SMN2* gene. The EAG considered that cost-effectiveness decisions should be made based on number of copies of the *SMN2* gene due to the following.

- Model results showed that QALYs and BSC costs differed substantially by number of copies of the *SMN2* gene. Patients with two copies of the *SMN2* gene have a higher likelihood of having type 1 SMA than patients with three copies of the *SMN2* gene. Furthermore, patients with type 1 SMA with three copies of the *SMN2* gene tend to have longer survival than those with two copies of the *SMN2* gene.
- Patients with two copies of the *SMN2* gene and those with three copies of the *SMN2* gene are identified at the time of diagnosis of SMA.
- Approximately 85% of patients with three copies of the *SMN2* gene have type 2 SMA (54.3%) or type 3 SMA (30.9%), not type 1 SMA (14.7%) [6], and therefore are not eligible for treatment with onasemnogene abeparvovec following the development of symptoms based on the recommendations made by NICE in HST15 [2].

The EAG therefore generated scenario results independently for patients with two copies of the *SMN2* gene and patients with three copies of the *SMN2* gene. The EAG scenario analyses explored two areas of uncertainty, namely loss of milestones achieved and social care costs.

In the company model, patients in the onasemnogene abeparvovec arm were modelled to maintain the best milestone they achieved, while, over time, patients in the BSC arm could lose milestones previously achieved. Milestone data were available from the SPR1NT trial for a maximum follow-up of 24 months, and from the START [21] trial for 6.2 years. This means that there is still uncertainty whether, over a lifetime, patients treated with onasemnogene abeparvovec would lose previously achieved milestones. To explore the impact of this uncertainty on company cost-effectiveness results, the EAG ran a scenario analysis applying the company base-case loss of milestone assumptions for the BSC arm of the long-term model to patients in the onasemnogene abeparvovec arm of the long-term model.

In the company model, social care costs accounted for the largest proportion of total costs after hospitalisations. The EAG highlighted there was uncertainty to how the company calculated social care costs as the value in the model did not match the costs presented in the publication by Noyes et al. [41]. To test the impact of these costs on company cost-effectiveness results, the EAG carried out a scenario in which the costs of social care were set to zero. The EAG considered that patients with SMA were likely to rely heavily on social care and that setting social care costs to zero is an extreme scenario; however, this scenario was undertaken to explore whether reducing social care costs would change the conclusions that can be drawn from the company’s cost-effectiveness results.

The EAG’s scenario cost-effectiveness results were generated for both the company’s preferred comparison and the EAG’s preferred comparison. All EAG scenario analyses generated ICERs for pre-symptomatic treatment with onasemnogene abeparvovec that were less than £100,000 per QALY gained.

### Conclusions of the EAG Report

The EAG considered that results from the SPR1NT trial supported the company’s conclusion that onasemnogene abeparvovec is a clinically effective treatment for babies with pre-symptomatic SMA and two or three copies of the *SMN2* gene. Naïve comparisons of data from the SPR1NT trial, the PNCR [20] dataset, and other trials [21–23] that evaluated onasemnogene abeparvovec as a treatment for patients with symptomatic SMA, suggested that outcomes for patients treated pre-symptomatically with onasemnogene abeparvovec are better than outcomes for patients who receive (1) onasemnogene abeparvovec upon a clinical diagnosis of type 1 SMA; and (2) BSC only for any type of SMA. However, these naïve comparisons were not robust and there remained some uncertainty about the long-term efficacy of onasemnogene abeparvovec in clinical practice given it is unclear whether some deterioration may occur.

The EAG also concluded that it is important to consider patients with two copies of the *SMN2* gene and patients with three copies of the *SMN2* gene separately as outcomes for these two groups differ substantially.

For the comparison of pre-symptomatic onasemnogene abeparvovec versus onasemnogene abeparvovec on development of symptoms of type 1 SMA and BSC for all other types of SMA, the EAG considered that pre-symptomatic treatment with onasemnogene abeparvovec is likely to be dominant. For the comparison of pre-symptomatic onasemnogene abeparvovec versus BSC, the EAG considered that the ICER per QALY gained is likely to be less than £100,000. Although the long-term efficacy of onasemnogene abeparvovec and costs associated with social care provision to children with SMA remained uncertain, these uncertainties were considered unlikely to change the conclusions that could be drawn on the cost effectiveness of onasemnogene abeparvovec given pre-symptomatically.

## National Institute for Health and Care Excellence: Request for Additional Modelling

Following submission of the EAG report, the Evaluation Committee requested additional evidence from the company. The committee understood that the company’s model results only related to babies treated at 6 weeks of age or younger, as this was the patient population included in the SPR1NT trial. The committee requested that the company adjust their model to assume diagnosis of pre-symptomatic SMA at 1 year of age, as newborn screening is not currently available in the NHS and diagnosis may occur much later than 6 weeks. The age at diagnosis of 1 year was chosen as the committee recalled that the HST15 recommendation for the treatment of pre-symptomatic SMA (via a managed access scheme) specified the population as ‘babies’, which NICE defines as those who are 1 year of age and younger. The committee noted in their request that results from an analysis that assumes diagnosis of pre-symptomatic SMA at 6 months of age would also be informative, as after this timepoint a diagnosis of type 1 SMA in the BSC arm would not be possible.

In their response to the NICE committee’s request, the company noted that a diagnosis of pre-symptomatic SMA after 6 weeks of age is rare. The company understood that there were two elements of the committee’s request for additional modelling to address.

1. Evaluating the cost effectiveness of treating patients with pre-symptomatic SMA with onasemnogene abeparvovec diagnosed later than 6 weeks, at up to 1 year of age.
2. Evaluating the cost effectiveness of treating patients with pre-symptomatic SMA with onasemnogene abeparvovec diagnosed by 6 weeks of age but not receiving treatment until after this timepoint

To address the first element, the company evaluated the cost effectiveness of onasemnogene abeparvovec for babies who are pre-symptomatic and aged 6 months and over at diagnosis and treatment. The company recalculated the probabilities of developing type 2 SMA and type 3 SMA (a diagnosis of type 1 SMA would not be possible for babies aged 6 months and over at diagnosis) for patients who receive BSC only based on clinical expert opinion of the proportion of patients with each *SMN2* copy number. The company also provided results from analyses that assumed patients in the BSC arm had equal probabilities of developing type 2 SMA and type 3 SMA. Furthermore, the company acknowledged that the efficacy of onasemnogene abeparvovec in babies aged 6 months and over may be reduced (in comparison with the efficacy of treating babies aged 6 weeks or younger) due to irreversible motor neurone damage, and therefore carried out analyses that assumed (1) no loss of efficacy, and (2) a reduction of 20% in the number of patients who would be able to walk for babies with two *SMN2* gene copies, and a reduction of 10% for babies with three *SMN2* gene copies (based on expert clinical opinion).

To address the second element, the company estimated cost-effectiveness results for patients who had a diagnosis of pre-symptomatic SMA before 6 weeks, but who did not receive treatment until after this timepoint. The company modelled treatment delays of 2, 4 and 6 weeks, and assumed a reduction in treatment efficacy based on clinical expert opinion.

As requested by the NICE committee, the EAG provided a short critique of the additional modelling undertaken by the company. The EAG was satisfied with the approach taken by the company to evaluate cost effectiveness for each scenario. However, the EAG considered the results for the second scenario (assuming diagnosis before 6 weeks of age, and a short delay in treatment) to be pessimistic, which suggested that if treatment does not commence by 22 weeks for patients with two copies of the *SMN2* gene, then these children would never walk (i.e., be type 2 SMA). The evidence presented in HST15 was that a proportion of symptomatic patients with two copies of the *SMN2* gene, if treated before 6 months, would be able to walk.

## National Institute for Health and Care Excellence: Evaluation Committee Meeting

In addition to the evidence presented by the company (Sects. 3.1, 3.3 and 4) and the EAG critique (Sects. 3.2, 3.4, 3.5 and 4), the NICE Evaluation Committee considered the views of patients and clinical experts. The committee made several observations following discussions with the patient and clinical experts, the company and the EAG at the Evaluation Committee meeting. The key points raised are summarised below.

### Consideration of the Clinical-Effectiveness Issues

The committee noted that onasemnogene abeparvovec is now recommended as a treatment option for patients with symptomatic type 1 SMA in HST15. Therefore, the committee concluded that the most relevant comparators for this evaluation were (1) onasemnogene abeparvovec for type 1 SMA, and (2) BSC for type 2 SMA and type 3 SMA.

The committee discussed the design, methods and results of the SPR1NT trial, noting that only a small number of babies were included in the trial, and the lack of long-term evidence available. Despite these limitations, the committee concluded that the results from the SPR1NT trial suggested that onasemnogene abeparvovec is effective in treating pre-symptomatic SMA.

The committee noted the SPRINT trial excluded patients who were over 6 weeks of age at commencement of treatment, yet in practice, diagnosis and/or treatment may occur much later than 6 weeks. Clinical experts explained that this may occur due to a delay in getting the results of a genetic test or contraindications such as elevated levels of adeno-associated virus serotype 9 (AVV9) antibodies (which may reduce over time and allow later treatment with onasemnogene abeparvovec). Clinical experts also highlighted that newborn screening is not currently available in the NHS, and routine pre-symptomatic testing for SMA only occurs when a sibling has SMA. This may also lead to babies with pre-symptomatic SMA being diagnosed when they are over 6 weeks of age. Clinical experts stated that onasemnogene abeparvovec would still be expected to provide important clinical benefits in babies with pre-symptomatic SMA aged over 6 weeks, but that the delay in starting treatment may lead to loss in efficacy compared with starting treatment earlier due to irreversible motor neuron damage. The committee considered the effect of age at treatment in its decision making by examining the results of additional modelling provided by the company (see Sect. 4).

### Consideration of the Cost-Effectiveness Issues

The committee noted that the company’s model assumed no motor milestone loss for patients treated with onasemnogene abeparvovec, and that there were limited long-term data to support this assumption. The committee agreed that this assumption was reasonable, as it was considered by the HST15 committee to be appropriate and in line with clinical expert opinion. The committee concluded that the company’s model was appropriate for decision making.

The committee heard from clinical experts that for babies and children treated with onasemnogene abeparvovec, the risk of experiencing AEs increases with age. The committee discussed the statement made by NHS England (shared with the UK SMA community in December 2022 [42]), which explained that there had recently been some serious AEs related to onasemnogene abeparvovec use in the NHS, particularly in older and heavier babies and children. These AEs led to NHS England placing a temporary pause on treatment with onasemnogene abeparvovec in children older than 12 months of age. The committee was concerned that the possible loss of QALYs due to AEs, and increased costs of treating AEs, were not included in the company’s model.

The committee considered the analyses provided by the company in response to NICE’s request for additional modelling. For the company’s first scenario analysis (evaluating the cost effectiveness of onasemnogene abeparvovec for babies aged ≥ 6 months at diagnosis and treatment of pre-symptomatic SMA), the NICE lead team considered the assumption of patients in the BSC arm having equal probabilities of developing type 2 SMA and type 3 SMA to be most reflective of a child aged 12 months, and therefore most relevant to the committee’s request for additional modelling. However, the company heard from one clinical expert who stated that a child aged 12 months with pre-symptomatic SMA would be more likely to develop type 3 SMA than type 2 SMA. In general, the committee concluded that the company’s additional scenario analyses were uncertain due to a lack of clinical data to inform them, but were still informative when considering onasemnogene abeparvovec as a treatment for babies aged over 6 weeks.

Clinical experts at the committee meeting confirmed that number of *SMN2* gene copies is the most useful factor for predicting outcomes in babies with pre-symptomatic SMA, but noted that even within subgroups defined by *SMN2* copy number, there remained uncertainty about expected outcomes. The committee considered the cost-effectiveness results by number of *SMN2* gene copies provided by the company to be informative, as incremental health benefits and costs are expected to vary for these groups.

The committee discussed the discount rate for costs and effects in this evaluation. NICE’s health technology evaluations manual [43] specifies that a discount rate of 1.5% may be used (instead of 3.5%) when treatment restores people to full or near-full health when they would otherwise die or have severely impaired lives, and benefits are likely to be sustained over a very long period. It was noted that a 1.5% discount rate had been accepted by the committee in HST15. However, in this partial review of HST15, the comparator patient population may go on to develop a range of SMA types, with a wide range of outcomes (whereas in HST15, symptomatic patients in the comparator all had type 1 SMA, and therefore would be expected to have particularly poor outcomes). Furthermore, onasemnogene abeparvovec is now routinely available for most babies who develop type 1 SMA, further reducing the gap between expected outcomes in the intervention (pre-symptomatic onasemnogene abeparvovec) and comparator arms (1) onasemnogene abeparvovec for type 1 symptomatic SMA, and (2) BSC for type 2 and type 3 SMA. The committee therefore concluded that it would not be appropriate to use a 1.5% discount rate in this evaluation, while also noting that decision making was not sensitive to the choice of discount rate used.

A most plausible incremental cost-effectiveness ratio (ICER) of below £100,000 per QALY gained for an HST is normally considered an effective use of NHS resources [44]. The committee noted that the company’s and EAG’s base-case analyses, the *SMN2* gene copy number subgroup analysis, and the EAG’s scenario analyses all indicated that onasemnogene abeparvovec for pre-symptomatic SMA dominated (1) onasemnogene abeparvovec for type 1 SMA, and (2) BSC for type 2 SMA and type 3 SMA. Considering the additional cost-effectiveness modelling requested by NICE, the committee’s preferred scenario, which assumed an equal chance of developing type 2 SMA and type 3 SMA for patients receiving BSC, generated ICER estimates below £100,000 per QALY gained. The committee concluded that onasemnogene abeparvovec was likely to be a cost-effective option for treating pre-symptomatic SMA in babies aged 12 months or under.

### Final Guidance

The committee recommended onasemnogene abeparvovec as an option for treating pre-symptomatic 5q SMA with a bi-allelic mutation in the *SMN1* gene and up to three copies of the *SMN2* gene in babies aged 12 months and under. It is only recommended if the company provides it according to the commercial arrangement (i.e. simple discount PAS).

## Conclusion

The primary source of evidence for this HST evaluation came from a small, single-arm trial [17, 18]; limited safety and long-term efficacy data were available. The committee considered that this trial was not fully generalisable to NHS clinical practice, as all babies included in the trial population were 6 weeks or younger. However, cost-effectiveness analyses conducted by the company and the EAG used a discounted price for onasemnogene abeparvovec and explored a range of different assumptions; all analyses generated ICERs that were less than £100,000 per QALY gained. Therefore, the committee concluded that pre-symptomatic treatment with onasemnogene abeparvovec would be a cost effective use of NHS resources.

### Supplementary Information

Below is the link to the electronic supplementary material.

Supplementary file1 (DOCX 19 KB)
