# Supplementary material for: Onasemnogene Abeparvovec for Treating Pre-symptomatic Spinal Muscular Atrophy: An External Assessment Group Perspective of the Partial Review of NICE Highly Specialised Technology Evaluation 15
Source: Pharmacoecon Open. 2023 Sep 20;7(6):863–75. doi: 10.1007/s41669-023-00439-6 (PMC10721753; doi:10.1007/s41669-023-00439-6)
Supplement: Supplementary file 1 — Supplementary file1 (DOCX 19 KB) [file 41669_2023_439_MOESM1_ESM.docx]

### PharmacoEconomics - Open

### Onasemnogene abeparvovec for treating pre-symptomatic spinal muscular atrophy: an external assessment group perspective of the partial review of NICE highly specialised technology evaluation 15

Marty Chaplin*, Rebecca Bresnahan, Nigel Fleeman, James Mahon, Rachel Houten, Sophie Beale, Angela Boland, Yenal Dundar, Ashley Marsden, Pinki Munot

*Corresponding author:

Marty Chaplin, Senior Research Fellow, Liverpool Reviews and Implementation Group, University of Liverpool

Email: [m.chaplin@liverpool.ac.uk](mailto:m.chaplin@liverpool.ac.uk)

### Funding

This project was funded by the National Institute for Health Research Evidence Synthesis Programme as project number NIHR135653. See the Evidence Synthesis Programme website for further project information. <https://www.nihr.ac.uk/explore-nihr/funding-programmes/evidence-synthesis.htm>.

The views and opinions expressed herein are those of the authors and do not necessarily reflect those of the National Institute for Health and Care Excellence or the Department of Health.

### Conflicts of interest

None of the authors (Marty Chaplin, Rebecca Bresnahan, Nigel Fleeman, James Mahon, Rachel Houten, Sophie Beale, Angela Boland, Yenal Dundar, Ashley Marsden, Pinki Munot) have any conflicts of interest to declare.

Supplementary Table 1 Definitions for PNCR dataset outcomes

| Cohort | Outcome and definition^a^ |
| --- | --- |
| Cohort with type 1 SMA and two copies of the SMN2 gene (n=23) | - Sits without support (full definition not available to the EAG) - Stands without support (full definition not available to the EAG) - Walk alone (full definition not available to the EAG) - Event-free survival, defined as avoidance of death or the requirement of permanent ventilation in the absence of acute illness or perioperatively at 14 months of age |
| Cohort with any type of SMA and three copies of the SMN2 gene (n=81) | - Ability to stand without support for at least 3 seconds (full definition marked as confidential) - Walk alone with coordination (full definition marked as confidential) - Event-free survival, defined as avoidance of death or the requirement of permanent ventilation in the absence of acute illness or perioperatively at 14 months of age |

SMA=spinal muscular atrophy; *SMN2*=survival motor neuron 2

Source: EAG report, Table 15
